# A95 INCIDENCE OF LUMINAL GASTROINTESTINAL CANCERS IN PATIENTS WITH CIRRHOSIS: A SYSTEMATIC REVIEW AND META-ANALYSIS

**DOI:** 10.1093/jcag/gwad061.095

**Published:** 2024-02-14

**Authors:** M Jogendran, K Zhu, R Jogendran, N Sabrie, D Chahal

**Affiliations:** Queen's University, Kingston, ON, Canada; The University of British Columbia Faculty of Medicine, Vancouver, BC, Canada; University of Toronto, Toronto, ON, Canada; University of Toronto, Toronto, ON, Canada; The University of British Columbia Faculty of Medicine, Vancouver, BC, Canada

## Abstract

**Background:**

The global incidence of cirrhosis is increasing, as is the incidence of luminal gastrointestinal cancers. It is unknown, however, if cirrhosis itself is a predisposing factor for luminal gastrointestinal cancer. Such an association would have significant clinical implications, particularly for cancer screening prior to liver transplantation.

**Aims:**

To investigate the incidence of luminal gastrointestinal cancers in patients with underlying cirrhosis.

**Methods:**

An electronic search was conducted to study the incidence of luminal gastrointestinal cancers in patients with cirrhosis. Study-specific standardized incidence ratios (SIR) along with their corresponding 95% confidence intervals for both overall cancer incidence and luminal cancer incidence were analyzed using a random-effects model. Statistical heterogeneity was assessed using the Cochran’s Q test and I2 statistic. Subgroup analysis was performed based on cirrhosis etiology and location of luminal malignancy.

**Results:**

We identified 5054 articles; 4 studies were selected for data extraction. The overall incidence of all cancers was significantly higher in patients with cirrhosis, with a SIR of 2.79 (95% CI 2.18–3.57). When stratified by cirrhosis etiology, the incidence of all cancers remained significantly elevated for alcohol (SIR 3.13, 95% CI 2.24–4.39), PBC (SIR 1.40, 95% CI 1.10–1.79), and unspecified cirrhosis (SIR 3.52, 95% CI 1.87–6.65). Unspecified cirrhosis had the greatest overall cancer risk followed by alcohol, and PBC cirrhosis (*p* ampersand:003C 0.01). Alcohol related cirrhosis was significantly associated with an increased incidence of luminal cancers (SIR 4.39, 95% CI 2.84–6.78). Specifically, the highest incidence was observed for oral cavity and pharyngeal cancer (SIR 10.44, 95% CI 8.90–12.24), followed by esophageal cancer (SIR 7.85, 95% CI 4.98–12.36), and colorectal cancer (SIR 2.43, 95% CI 1.62–3.66) (*p* ampersand:003C 0.01). PBC cirrhosis was significantly associated with an increased incidence of luminal cancers (SIR 2.11, 95% CI 1.10–4.04). However, subgroup analysis did not reveal a significant association with the incidence of any subgroups. Unspecified cirrhosis was significantly associated with an increased incidence of luminal cancers (SIR 2.60, 95% CI 1.65–4.09). Risk of oral cavity and pharynx cancers (SIR 4.75, 95% CI 2.18–10.33) were greatest, followed by esophageal (SIR 4.52, 95% CI 1.57–12.99), stomach (SIR 2.12, 95% CI 1.31–3.42), and colorectal cancer (SIR 1.57, 95% CI 1.14–2.16) (*p* = 0.03).

**Conclusions:**

The incidence of luminal gastrointestinal cancer is increased amongst patients with cirrhosis. Oral cavity, pharyngeal and esophageal cancer had increased incidence across all cirrhosis etiologies compared to gastric and colorectal cancer. Therefore, increased screening of luminal cancers should be considered in this population.

**Funding Agencies:**

None

